# Treatment of peritoneal disease arising from mucinous vs. non-mucinous appendiceal neoplasms with cytoreductive surgery and hyperthermic intraperitoneal chemotherapy

**DOI:** 10.1515/pp-2025-0007

**Published:** 2025-07-23

**Authors:** Rennie Xinrui Qin, Tilisi Puloka, Jia Hui Lim, Caro Staheli, Jesse Fischer, Simione Lolohea, Jasen Ly

**Affiliations:** Department of General Surgery, Te Whatu Ora Waikato 3714 , Hamilton, New Zealand; Department of Epidemiology and Biostatistics, Faculty of Medical and Health Sciences, University of Auckland, Auckland, New Zealand; Waikato Clinical Campus, Faculty of Medical and Health Sciences, University of Auckland, Auckland, New Zealand

**Keywords:** appendiceal neoplasms, cytoreductive surgery, hyperthermic intraperitoneal chemotherapy, non-mucinous appendiceal neoplasms, pseudomyxoma peritonei

## Abstract

**Objectives:**

Non-mucinous appendiceal neoplasms (NMAN) are rare. The role of cytoreductive surgery (CRS) and hyperthermic intraperitoneal chemotherapy (HIPEC) in treating peritoneal dissemination from NMAN is poorly defined. We hypothesise that histology impacts survival and compared the disease characteristics and short- and long-term outcomes of mucinous and non-mucinous appendiceal neoplasms treated with CRS/HIPEC.

**Methods:**

We retrospectively reviewed a prospective database of 228 patients with peritoneal disease from appendiceal primaries proceeding to CRS/HIPEC from 01/01/2008 to 30/06/2022 at a tertiary referral centre in New Zealand.

**Results:**

There were 209 mucinous appendiceal neoplasms (MANs) and 19 NMANs. NMANs were more likely to metastasise to lymph nodes (p<0.001) and be treated with systemic chemotherapy (p<0.001) than MANs. Surgery for NMAN was more likely to involve small bowel resection (p<0.001) and less likely to achieve complete cytoreduction (p<0.001). Short-term outcomes were similar between MAN and NMAN. CRS/HIPEC for NMAN had a major complication rate of 15.3 % and no perioperative mortality. Extraperitoneal recurrence, including pleural and systemic recurrence, was more likely to occur in NMAN than all grades of MAN. The median overall survival was not reached in MAN and 16.0 months in NMAN. High PCI, ECOG, and tumour grade were associated with poor survival in NMAN.

**Conclusions:**

The prognosis following CRS/HIPEC for NMAN is poor. Patients with NMAN need to be judiciously selected for CRS/HIPEC.

## Introduction

Appendiceal neoplasms are rare, with an annual incidence of up to 0.97 per 100,000 population [1]. They exhibit unique biological behaviour with a propensity for isolated peritoneal dissemination [2]. Mucinous appendiceal neoplasms (MANs) give rise to pseudomyxoma peritonei (PMP), a clinical syndrome characterised by mucinous ascites and solid or semi-solid nodular implants [2]. Over the last four decades, locoregional disease control through cytoreductive surgery (CRS) and hyperthermic intraperitoneal chemotherapy (HIPEC) has revolutionised the treatment of PMP and is the standard of care for mucinous peritoneal dissemination from appendiceal tumours worldwide [3].

A subgroup of appendiceal neoplasm remains poorly understood. Non-mucinous appendiceal neoplasms (NMANs) are even rarer and constitute less than 10 % of appendiceal neoplasms requiring CRS/HIPEC [4], 5]. NMANs have a higher propensity for systemic dissemination via haematogenous and lymphatic routes than MANs, comparable to adenocarcinoma and neuroendocrine tumours [6], 7]. Long-term oncological outcomes, including survival following treatment of NMAN with CRS/HIPEC, are inferior to their mucinous counterpart [4], 8]. Despite the wide adoption of CRS/HIPEC for peritoneal metastases of appendiceal origin, little evidence and no guidelines exist on the treatment of peritoneal disease specifically arising from NMAN. We hypothesise that histology affects survival and recurrence following CRS/HIPEC in appendiceal neoplasms. In order to assess this, we compared the difference in disease characteristics, management strategies, and long-term outcomes between mucinous and non-mucinous appendiceal neoplasms and between different histological sub-types of NMAN.

## Materials and methods

### Study population

This retrospective cohort study is based on a prospectively maintained database of all patients proceeding to CRS/HIPEC from 01/01/2008 to 30/06/2022 at a dual-site, single-service tertiary referral centre in Aotearoa New Zealand. Aotearoa New Zealand is a country of five million. The Waikato region accepts referrals from all regions of the country and provides CRS/HIPEC through Waikato Hospital in the public sector and Braemar Hospital in the private sector. All patients with disseminated peritoneal disease from an appendix primary who underwent surgery with the intention to perform CRS/HIPEC were included in the study.

### Patient management

Patient management pathways in Waikato have been described previously and are briefly summarised here [9]. All patients were discussed at a multidisciplinary meeting (MDM) and considered for CRS/HIPEC if they had no major organ dysfunction, good performance status, no unresectable distant metastases, and peritoneal disease amenable to complete cytoreduction on preoperative investigations. No peritoneal cancer index (PCI) restrictions were placed on peritoneal dissemination from an appendiceal primary. CRS/HIPEC was performed according to the standard Sugarbaker technique with mitomycin C administered via an open coliseum technique at 41–42 °C for 90 min when complete cytoreduction was achieved [10]. A PCI and completeness of cytoreduction (CC) score were calculated for each patient [11].

The pathological specimens from the CRS/HIPEC and the index operation at the referring hospital were routinely reviewed at the MDM. Routine postoperative surveillance consisted of four monthly tumour markers (CEA, CA19-9 and CA 125) and a computerised tomography (CT) of the chest, abdomen, and pelvis at 1, 2, 3, and 5 years. Long-term survival and recurrence data were collected from the most recent clinical or radiological follow-up at the referring centre and the Waikato region.

### Histology assessment

Primary appendiceal tumours and peritoneal disease were classified according to the Peritoneal Surface Oncology Group International (PSOGI) consensus [2]. Tumours were defined as mucinous when more than 50 % of the cross-sectional area histologically comprised extracellular mucin and as non-mucinous when this was not the case, according to the World Health Organization classification [12].

According to the PSOGI consensus, appendiceal tumours are classified as low-grade appendiceal mucinous neoplasm (LAMN), high-grade appendiceal mucinous neoplasm (HAMN), mucinous adenocarcinoma (MA), intestinal adenocarcinoma of the colorectal type (ITAC), goblet cell adenocarcinoma (GCA), and neuroendocrine tumour [2]. GCA and mixed adenoneuroendocrine carcinoma (MANEC) have features of both adenocarcinoma and neuroendocrine tumours [12]. GCA was previously known as goblet cell carcinoid or adenocarcinoid. GCA is defined by well-differentiated goblet cells with positive epithelial (CK20, CDX2, CD46) and neuroendocrine (synaptophysin and chromogranin) staining [12]. MANECs display high-grade and poorly differentiated neuroendocrine features with ki-67>20 % [12]. GCA and MANEC can be mucinous or non-mucinous. The mucinous disseminated peritoneal disease is characterised by mucinous ascites and peritoneal implants and is classified as (1) acellular mucin (AC), (2) low-grade mucinous carcinoma peritonei (LG), (3) high-grade mucinous carcinoma peritonei (HG), and (4) high-grade mucinous carcinoma peritonei with signet ring cells (HG-S) [2].

### Statistical analysis

Descriptive statistics are reported as frequencies and proportions for categorical variables and median and interquartile range for continuous variables. Statistical testing for baseline characteristics was conducted using the Kruskal–Wallis rank sum test, Wilcoxon rank sum test, Chi-square test, and Fisher’s exact test as appropriate. Overall survival (OS) and disease-free survival (DFS) were assessed using Kaplan–Meier survival analysis. Survival and recurrence were determined from the time of procedure to the time of death and recurrence, respectively. Patients were censored at their last follow-up date. Factors affecting survival were identified using univariate and multivariate Cox proportional hazard models. Significant factors in the univariate analysis were entered into the multivariate model. p-values were two-sided, with statistical significance evaluated at the 0.05 alpha level. Statistical analyses were conducted using R Studio version 2023.03.0 + 386 for Mac.

### Ethics consideration

This study was approved by the New Zealand Health and Disability Ethics Committee (HDEC 2023 FULL 15566) on 23/05/2023 and was conducted in accordance with the Declaration of Helsinki.

## Results

A total of 228 patients had disseminated peritoneal disease from an appendiceal primary tumour. Table 1 displays their baseline characteristics. Two cases were presumed to arise from an appendix primary, as the appendix was suspected of being replaced by malignancy, and no other primaries were found on endoscopy, radiology or operative histology.

**Table 1:** Demographic and clinical characteristics of 228 patients with disseminated peritoneal disease of appendiceal origin.

Characteristic	Categories	Non-mucinous N=19	Mucinous N=209	p-Value
Gender	Male	7 (36.8 %)	96 (45.9 %)	0.48
	Female	12 (63.2 %)	113 (54.1 %)	
Age	Median (IQR)	54.0 (51.0, 64.0)	56.0 (48.0, 65.0)	0.72
Māori & Pacific ethnicity	Yes	1 (5.3 %)	51 (24.4 %)	0.083
Previous surgery	Yes	15 (78.9 %)	125 (59.8 %)	0.11
Previous chemotherapy	Yes	7 (36.8 %)	18 (8.6 %)	0.002**
Elevated CEA (>5 ng/mL)^a^	Yes	7 (38.9 %)	89 (60.1 %)	0.085
Tumour	LAMN	0 (0 %)	156 (74.6 %)	<0.001***
	HAMN	0 (0 %)	21 (10.0 %)	
	MACA	0 (0 %)	29 (13.9 %)	
	ITAC	9 (47.4 %)	0 (0 %)	
	GCA	8 (42.1 %)	1 (0.5 %)	
	MANEC	2 (10.5 %)	0 (0 %)	
	Replaced	0 (0 %)	2 (1.0 %)	
Lymph nodes sampled	Yes	10 (52.6 %)	113 (54.1 %)	1.00
Lymph nodes involved^b^	Median (IQR)	2 (0, 2)	0 (0, 0)	<0.001***
Lymph node positivity^b^	Yes	7 (70.0 %)	9 (8.0 %)	<0.001***
Grade	Median (IQR)	3 (2, 3)	1 (1, 1)	<0.001***

n (%); Median (IQR); Pearson’s chi-squared test; Wilcoxon rank sum test; Fisher’s exact test. CEA, carcinoembryonic antigen; GCA, goblet cell adenocarcinoma; HAMN, high-grade appendiceal mucinous neoplasm; IQR, interquartile range; ITAC, intestinal adenocarcinoma of the colorectal type; LAMN, low-grade appendiceal mucinous neoplasm; MA, mucinous adenocarcinoma; MANEC, mixed adenoneuroendocrine carcinoma. ^a^Data available in 18 non-mucinous cases and 148 mucinous cases. ^b^Data available in 10 non-mucinous cases and 113 mucinous cases. *p<0.05, **p<0.01, ***p<0.001.

### Appendiceal non-mucinous neoplasms

Among 19 patients with NMANs, nine were ITAC, eight were GCA, and two were MANEC. Among the nine ITACs, five cases were moderately differentiated, and four were poorly differentiated. No cases were associated with signet ring cells (SRCs). The adenocarcinoma component of GCAs was moderately differentiated in three patients and poorly differentiated with SRC in four patients. Both cases of MANEC were poorly differentiated with SRC. Fifteen patients had previous surgery, including six appendicectomies and five right hemicolectomies. Lymph nodes were sampled in 10 patients and involved in seven patients. Seven patients received previous systemic chemotherapy.

Compared to MANs, NMANs were associated with a significantly higher tumour grade (p<0.001), more involved lymph nodes (p<0.001), and a higher proportion of positive lymph nodes (p<0.001) and systemic chemotherapy use (p=0.002).

### Appendiceal mucinous neoplasms

A total of 209 patients were diagnosed with MAN, including 156 LAMN, 21 HAMN, 29 MA, 1 GCA, and two replaced primaries. A total of 125 patients underwent previous surgery for MAN, including 68 appendicectomies and 46 right hemicolectomies, among other surgeries. Lymph nodes were sampled in 113 patients and involved in nine. Eighteen patients received previous systemic chemotherapy.

### Operative outcomes

Table 2 displays operative and early postoperative outcomes. Patients with HG and HG-S mucinous neoplasms were grouped as HG due to small numbers. The median PCI was lower in AC (4) compared to LG (23), HG (19), and NMAN (26) (p<0.001). The complete cytoreduction rate was 100 % (30/30) in AC, 87.7 % (114/130) in LG, 69.4 % (34/49) in HG, and 42.1 % (8/19) in NMAN (p<0.001). There was an associated decrease in HIPEC use in AC compared to NMAN (p<0.001). Eleven patients with NMAN had incomplete cytoreduction. Eight underwent palliative debulking and bypass, and three had open-and-close procedures. Complete cytoreduction was not achievable due to infiltrative small bowel and mesenteric disease in eight patients and extensive upper abdominal disease, including periportal and peri-gastric disease, in three patients.

**Table 2:** Operative and short-term outcomes in 228 patient with disseminated peritoneal disease of appendiceal origin.

Variable	AC, N=30	LG, N=130	HG, N=49	NMAN, N=19	p-Value
Peritoneal cancer index^a^	4 (3, 10)	23 (10, 33)	19 (9, 36)	26 (16, 30)	<0.001***
Complete cytoreduction	30 (100.0 %)	114 (87.7 %)	34 (69.4 %)	8 (42.1 %)	<0.001***
HIPEC use	30 (100.0 %)	113 (86.9 %)	32 (65.3 %)	8 (42.1 %)	<0.001***
Stoma formation	0 (0.0 %)	43 (33.1 %)	16 (32.7 %)	5 (26.3 %)	0.003**
Number of organs resected	3 (2, 4)	4 (3, 5)	3 (2, 4)	2 (1, 4)	<0.001***
Number of peritonectomies	2 (1, 3)	5 (2, 6)	2 (0, 5)	0 (0, 2)	<0.001***
RBC transfusions	0 (0, 0)	0 (0, 3)	0 (0, 2)	0 (0, 0)	0.002**
Duration	454 (83)	554 (160)	472 (205)	368 (199)	<0.001***
Length of stay	10 (7, 13)	12 (9, 15)	12 (8, 17)	10 (6, 14)	0.076
Major complications	5 (16.7 %)	35 (26.9 %)	12 (24.5 %)	3 (15.8 %)	0.60
Return to theatre	4 (13.3 %)	12 (9.2 %)	8 (16.3 %)	0 (0.0 %)	0.21
Perioperative mortality	0 (0.0 %)	3 (2.3 %)	0 (0.0 %)	0 (0.0 %)	0.79

Kruskal-Wallis rank sum test; Fisher’s exact test; Pearson’s chi-squared test. AC, acellular mucin; HIPEC, hyperthermic intraperitoneal chemotherapy; HG, high-grade mucinous carcinoma peritonei with and without signet cells; LG, low-grade mucinous carcinoma peritonei; NMAN, non-mucinous appendiceal neoplasm; RBC, red blood cell. ^a^Data available in 26 in AC, 121 in LG, 45 in HG, 18 in NM. *p<0.05, **p<0.01, ***p<0.001.

Stomas were significantly less likely to be formed in AC (0 %, 0/0) compared to LG (33.1 %, 43/130), HG (32.7 %, 16/49), and NMAN (26.3 %, 5/19) (p=0.003). The number of organs resected (p<0.001), the number of peritonectomies (p<0.001), operative duration (p<0.001), and red blood cell transfusions (p=0.002) were the highest in LG, followed by HG and AC, and the lowest in NMAN. A greater proportion of most organs were resected in patients with MAN than NMAN. However, the small bowel was resected in 36.8 % (7/19) of NMAN compared to 9.6 % (20/209) of mucinous disease (p=0.003).

When the analysis was confined to cases with complete cytoreduction, patients with mucinous and non-mucinous disease had a similar PCI (14 vs. 17, p=0.6), lower than the main cohort. There were no longer differences between the two groups in organ resection, peritonectomy, duration, and transfusion.

Short-term outcomes were comparable between the mucinous and non-mucinous groups. Within the NMAN group, there were three cases of major complications (15.8 %, 3/19), no return to theatre (0 %, 0/19), or perioperative mortality (0 %, 0/19).

### Survival

**Figure 1:**
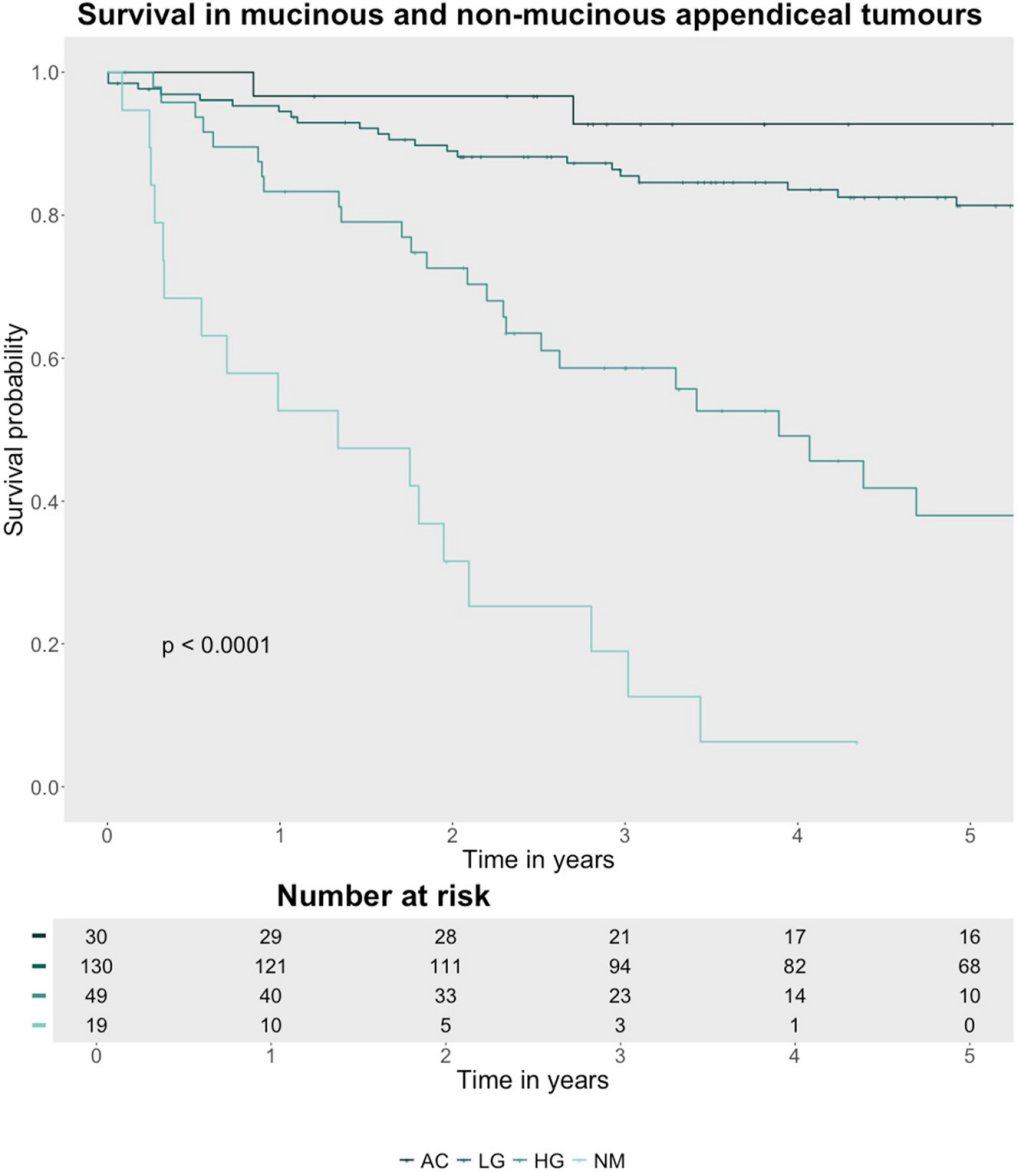
Overall survival in patients with peritoneal dissemination from mucinous and non-mucinous appendiceal neoplasms. AC: acellular mucin; LG: low-grade carcinoma peritonei; HG: high-grade mucinous carcinoma peritonei with and without signet cells; NMAN: non-mucinous appendiceal neoplasm.

Median follow-up was 16 months in NMAN and 53 months in MAN. Seventy-two patients died. The cause of death was disease progression in 62 patients, unrelated causes in six patients, treatment side effects in three patients, and unknown in one patient. NMAN had significantly poorer OS (Figure 1). The median OS was not reached in MAN and 16.0 months in NMAN. Among MAN, the median OS was not reached in AC and LG and 46.6 months in HG (Table 3). 5-year survival was 92.8 % in AC, 81.4 % in LG, 38.0 % in HG, and 6.3 % in NMAN (p<0.001).

**Table 3:** Overall survival in 228 patients with disseminated peritoneal disease of appendiceal origin.

	N	Events	Median OS	2-year OS	5-year OS	Hazard ratio	p-Value
AC	30	2	NA	96.6 % (90.4–100 %)	92.8 % (83.6–100 %)	NA	<0.001***
LG	130	27	NA	89.0 % (83.7–94.6 %)	81.4 % (74.6–88.8 %)	3.1	
HG	49	29	46.6	72.6 % (60.9–86.5 %)	38.0 % (24.7–58.5 %)	12.8	
NMAN	19	17	16.0	31.6 % (16.3–61.2 %)	6.3 % (1.0–41.1 %)	40.4	

AC, acellular mucin; HG, high-grade mucinous carcinoma peritonei with and without signet cells; LG, low-grade mucinous carcinoma peritonei; NMAN, non-mucinous appendiceal neoplasm; N, patient number; OS, overall survival. *p<0.05, **p<0.01, ***p<0.001.

**Figure 2:**
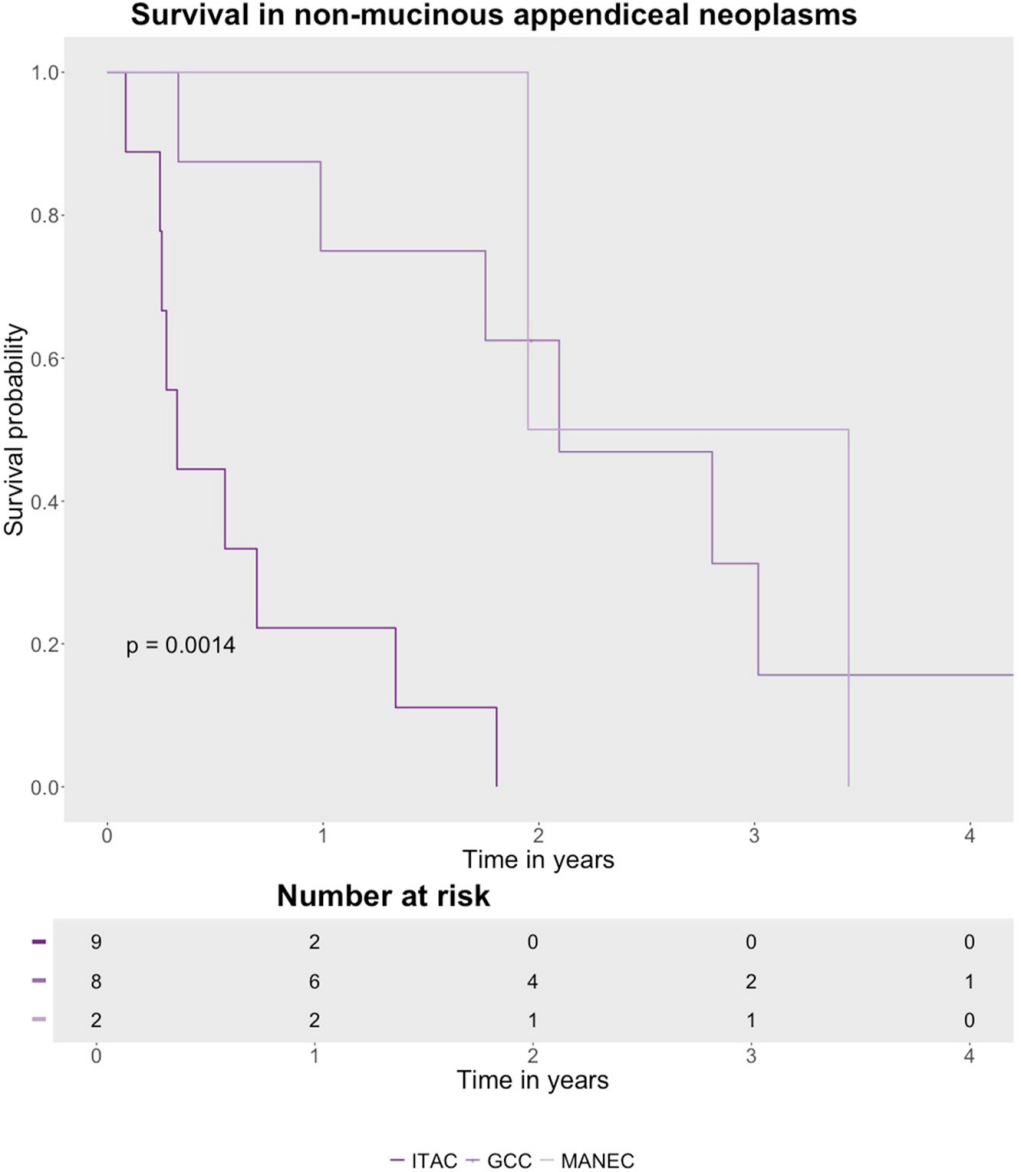
Overall survival in patients with peritoneal dissemination from non-mucinous appendiceal neoplasms. GCA: goblet cell adenocarcinoma; ITAC: intestinal adenocarcinoma of the colorectal type; MANEC: mixed adenoneuroendocrine carcinom.

Within the NMAN subgroup, ITAC had the poorest survival (Figure 2, p=0.001). Among nine patients with ITAC, only two patients had complete cytoreduction, and both recurred. Eight patients with ITAC died with a median OS of 3.9 months. Among eight patients with GCA, the median OS was 25.1 months. Six had complete cytoreduction; all recurred. Both patients with MANEC had incomplete cytoreduction and died with a median OS of 32.2 months. NMAN patients who had complete cytoreduction had a median OS of 25.1 months.

On univariate analysis, poor survival across the entire cohort was associated with advanced age (p=0.008), American Society of Anaesthesiologists score>1 (p=0.025), high European Cooperative Oncology Group (ECOG) performance status (p<0.001), previous systematic chemotherapy (p=0.001), high PCI (p<0.001), high grade (p<0.001), incomplete cytoreduction (p<0.001) and tumour type (p<0.001), the presence of SRC (p<0.001), and lymph node involvement (p<0.001) (Table 4). Lymph node involvement was not included in the multivariate model due to a large number of missing data. Incomplete cytoreduction (p<0.001) and NMAN (p=0.041) remained significant predictors in multivariate analysis. In a separate model for NMAN patients alone, high PCI (p=0.02), ECOG (p=0.004), and high tumour grade (p=0.005) were risk factors for poor survival in NMAN.

**Table 4:** Factors associated with overall survival in 228 patients with disseminated peritoneal disease of appendiceal origin.

Characteristic		Univariate			Multivariate		
		HR	95 % CI	p-Value	HR	95 % CI	p-Value
Age		1.03	1.01, 1.05	0.008**	1.03	0.99, 1.05	0.079
Gender		0.67	0.42, 1.06	0.08			
Maori & pacific ethnicity		0.90	0.52, 1.57	0.72			
ASA>1		2.08	1.10, 3.95	0.025*	1.36	0.62, 2.99	0.44
ECOG		3.12	1.97, 4.93	<0.001***	1.29	0.74, 2.26	0.37
Systemic chemotherapy		2.56	1.43, 4.59	0.001**	1.11	0.49, 2.50	0.80
Prior surgical score		0.83	0.52, 1.31	0.42			
Elevated CEA		1.26	0.76, 2.10	0.37			
PCI		1.06	1.03, 1.08	<0.001***	1.02	0.99, 1.04	0.24
Complete cytoreduction		0.06	0.04, 0.11	<0.001***	0.15	0.07, 0.29	<0.001***
Transfusion		0.90	0.56, 1.43	0.65			
Tumour type	AC	–	–		–	–	
	LG	3.14	0.75, 13.2	0.12	1.50	0.33, 6.80	0.60
	HG	12.8	3.06, 53.9	<0.001***	3.16	0.59, 16.89	0.18
	NMAN	40.4	9.20, 177	<0.001***	7.89	1.09, 57.20	0.041*
Signet ring cells		3.79	2.19, 6.56	<0.001***	0.76	0.31, 1.86	0.54
Grade		2.51	1.92, 3.27	<0.001***	1.24	0.68, 2.28	0.49
Lymph node involvement^a^		5.92	2.75, 12.	<0.001***	–	–	–

AC, acellular mucin; ASA, American society of anaesthesiologists; CEA, carcinoembryonic antigen; CI, confidence interval; ECOG, European cooperative oncology group; HG, high-grade mucinous carcinoma peritonei with and without signet cells; HR, hazard ratio; LG, low-grade mucinous carcinoma peritonei; NM: non-mucinous tumours; PCI: peritoneal cancer index. ^a^Data available in 223 patients. *p<0.05, **p<0.01, ***p<0.001.

### Recurrence

Recurrence occurred in 68 out of 186 patients with complete cytoreduction. The site of recurrence was peritoneal in 62 patients, systemic in 13 patients, and pleural in eight patients. Peritoneal recurrence, was more likely to occur in NMAN, HG, and LG, compared to AC (p=0.001) (Table 5). Extraperitoneal recurrence, including pleural and systemic recurrence, was more likely to occur in NMAN than HG, LG, and AC. Among six GCAs that received complete cytoreduction, recurrence occurred in the peritoneal cavity in four patients, the kidney in one patient, and the lung in one patient. Both cases with ITAC recurred in the peritoneal cavity; one also developed pleural and lung metastases. Following complete cytoreduction, DFS was significantly worse in NMAN compared to HG, LG, and AC (p<0.001) (Table S1).

**Table 5:** Recurrence following cytoreductive surgery and hyperthermic intraperitoneal chemotherapy in 228 patients with disseminated peritoneal disease of appendiceal origin.

Characteristic	AC, N=30	LG, N=130	HG, N=49	NMAN, N=19	p-Value
Recurrence	1 (3.3 %)	38 (29.2 %)	23 (46.9 %)	8 (42.1 %)	<0.001***
Peritoneal recurrence	1 (3.3 %)	36 (27.7 %)	22 (44.9 %)	7 (36.8 %)	<0.001***
Extraperitoneal recurrence	0 (0.0 %)	4 (3.1 %)	5 (10.2 %)	4 (21.1 %)	0.006*
Pleural recurrence	0 (0.0 %)	3 (2.3 %)	5 (10.2 %)	3 (15.8 %)	0.009*
Systemic recurrence	0 (0.0 %)	0 (0.0 %)	4 (8.2 %)	2 (10.5 %)	0.002*

Fisher’s exact test. AC, acellular mucin; HG, high-grade mucinous carcinoma peritonei with and without signet cells; LG, low-grade mucinous carcinoma peritonei; NMAN, non-mucinous appendiceal neoplasm. *p<0.05, **p<0.01, ***p<0.001.

## Discussion

This study found substantial differences in disease characteristics, operative management, and prognosis between peritoneal dissemination from mucinous and non-mucinous appendiceal neoplasms treated with CRS/HIPEC. NMANs were more likely to metastasise to lymph nodes and develop extraperitoneal recurrence. CRS/HIPEC for NNAM was less likely to achieve complete cytoreduction. Survival was significantly poorer in non-mucinous compared to mucinous appendiceal neoplasms.

Due to the rarity of NMAN, only a few studies with small numbers have examined the treatment of peritoneal dissemination from NMAN with CRS/HIPEC. Previous studies have detected similar differences in disease characteristics between mucinous and non-mucinous appendiceal tumours [4], 7]. Studies reported that NMAN had more involved lymph nodes, a lower proportion of complete cytoreduction, and more systemic recurrences consistent with the reported findings of the current study [4], 7]. A study examining appendiceal adenocarcinoma using the Surveillance, Epidemiology, and End Results database found that CRS benefited mucinous but not non-mucinous appendiceal adenocarcinoma [13]. Previous studies have found poor survival in NMAN. Garach et al. reported a median OS of 24 months across NMAN subgroups [4].

Molecular factors may explain differences in the biological behaviour of mucinous and non-mucinous appendiceal tumours. MAN harbours more KRAS and GNAS mutations and fewer TP53 mutations [7]. They were characterised by the over-expression of mucin 2, a gel-forming mucus protein secreted in the small bowel and colon with a normal protective function [14]. This results in its propensity to accumulate in the peritoneum in the classic pattern of PMP. PMP with low-grade peritoneal disease has minimal cytological atypia and expansile growth of a pushing type, which can be particularly amenable to CRS/HIPEC [2]. Peritoneal disease in NMAN demonstrated more infiltrative and destructive invasion similar to conventional colorectal cancer and was more likely to involve the small bowel [15]. This is consistent with our findings of a higher proportion of small bowel resection in the NMAN group.

NMANs are histologically heterogeneous and consist of several subtypes with distinct tumour biology. Outcomes following CRS/HIPEC for GCA have been more widely reported in the literature. A systematic review identified nine retrospective cohort studies reporting a median OS of 17–27 months, consistent with the median OS of 25.1 months reported in this study [16]. Very few studies have reported long-term outcomes following CRS/HIPEC for ITAC. Two previous studies reported a median OS of 17 and 38.7 months in a cohort with a median PCI of 16, longer than the median OS of 3.9 months in the ITAC group in this study [4], 13]. This may be due to a higher median PCI of 26 in the NMAN group of this study than previous studies in the literature. This raises the need to further explore a PCI cut-off in patient selection in the NMAN group at our institution [4], 5], 16]. Our study also found that GCA had more favourable survival compared to ITAC. However, the results for the NMAN subgroup should be interpreted with caution due to the small numbers and variations in baseline characteristics resulting from the retrospective study design.

Although survival following CRS/HIPEC for NMAN in this study is poor, previous studies have argued for the role of CRS/HIPC in well-selected patients with NMAN [8]. Randomised control studies are not possible when studying surgical interventions for rare diseases due to impractically long accrual periods during which treatment paradigms change. Propensity score-matched analyses have found improved median OS from 12 to 39 months following CRS/HIPEC for GCA [16]. The literature has demonstrated that CRS/HIPEC is safe for NMAN [4], 16]. Given that NMANs have unique tumour biology compared to colorectal cancer, with a greater predilection for developing isolated peritoneal metastasis, it is essential to develop effective locoregional treatment for isolated peritoneal metastases from NMAN [6], 7].

Patient selection is crucial in the management of peritoneal disease from NMAN. Our study found low PCI, ECOG, and grade were associated with improved survival among NMANs. PCI has been established as an important criterion in patient selection for CRS in colorectal cancer [17]. However, no guidelines exist on patient selection for CRS/HIPEC in NMAN. Previous studies have demonstrated better survival in NMAN with PCI<15 and GCA with PCI<20 [4], 18]. In the literature, grade is a well-established prognostic factor in GCA [19], 20] and ITAC and correlates with the KRAS mutation [8], 21]. In addition, complete cytoreduction and no lymph node involvement have also been found to be predictive factors of survival in NMANs treated with CRS/HIPEC in the literature.

Although CRS/HIPEC has been practised for more than four decades and is the standard of care for mucinous peritoneal dissemination of appendiceal neoplasms, the evidence supporting its use for NMAN remains extremely limited. Given the poor outcomes reported in this study and the literature, CRS/HIPEC should be cautiously approached in peritoneal dissemination from NMAN.

Similar to previous studies on NMAN, our study was limited by its retrospective nature and small numbers due to its rarity. Despite a small sample size of NMAN, which increases type II error and reduces statistical power, our study was able to detect many statistically significant differences between MAN and NMAN. This likely reflects the substantial underlying differences between these histology types as outlined by previous literature [4]. Our study is also subject to systemic bias in addition to random error. Our cohort may differ in baseline characteristics from other centres and merely reflects single-centre practice. Furthermore, data in variables such as lymph node involvement and tumour marker levels were missing, limiting our ability to assess their prognostic utility. Our study is exploratory and hypothesis-generating. Future studies should further explore survival predictors in NMAN, such as a PCI cut-off and tumour grade for each NMAN tumour type, to inform optimal patient selection and treatment guideline development.

## Conclusions

Although NMANs share a predilection for developing isolated peritoneal metastases, they have distinct tumour behaviour compared to MAN, making them less amenable to complete cytoreduction with CRS/HIPEC. The prognosis following CRS/HIPEC for NMAN is poor. This exploratory study demonstrated that patients with NMAN need to be judiciously selected for CRS/HIPEC. Selection criteria for surgery based on PCI and clinic-pathological criteria should be developed by future studies.
